# Some new *k*-Riemann–Liouville fractional integral inequalities associated with the strongly *η*-quasiconvex functions with modulus $\mu\geq0$

**DOI:** 10.1186/s13660-018-1736-5

**Published:** 2018-06-20

**Authors:** Eze R. Nwaeze, Seth Kermausuor, Ana M. Tameru

**Affiliations:** 1 Department of Mathematics, Tuskegee University, Tuskegee, USA; 2 Department of Mathematics and Computer Science, Alabama State University, Montgomery, USA

**Keywords:** 26A51, 26D15, 26E60, 41A55, Hermite–Hadamard inequality, Strongly *η*-quasiconvex, Riemann–Liouville fractional integrals

## Abstract

A new class of quasiconvexity called strongly *η*-quasiconvex function was introduced in (Awan et al. in Filomat 31(18):5783–5790, 2017). In this paper, we obtain some new *k*-Riemann–Liouville fractional integral inequalities associated with this class of functions. For specific values of the associated parameters, we recover results due to Dragomir and Pearce (Bull. Aust. Math. Soc. 57:377–385, 1998), Ion (Ann. Univ. Craiova, Math. Sci. Ser. 34:82–87, 2007), and Alomari et al. (RGMIA Res. Rep. Collect. 12(Supplement):Article ID 14, 2009).

## Introduction

Let $I\subset \mathbb{R}$ be an interval, and let $I^{\circ }$ denote the interior of *I*. We say that a function $g:I\rightarrow \mathbb{R}$ is quasiconvex if

$$ g\bigl(tx+(1-t)y\bigr)\leq \max \bigl\{ g(x), g(y)\bigr\} $$

for all $x, y\in I$ and $t\in [0, 1]$.

For functions that are quasiconvex on $[a, b]$, Dragomir and Pearce [5] established the following inequality of the Hermite–Hadamard type.

### Theorem 1

*Let* $g:[a, b]\rightarrow \mathbb{R}$ *be a quasiconvex positive function*. *If* $g\in L_{1}([a,b])$, *then we have the following succeeding inequality*:

1 $$ \frac{1}{b-a} \int_{a}^{b}g(t)\,dt\leq \max \bigl\{ g(a), g(b)\bigr\} . $$

Ion [8] obtained the following two results in the same direction.

### Theorem 2

*Let* $g:[a, b]\rightarrow \mathbb{R}$ *be a differentiable function on* $(a, b)$. *If*, *in addition*, *the absolute value function* $\vert g' \vert $ *is quasiconvex on* $[a, b]$, *then we have the following succeeding inequality*:

2 $$ \biggl\vert \frac{g(a)+g(b)}{2}-\frac{1}{b-a} \int_{a}^{b}g(t)\,dt \biggr\vert \leq \frac{b-a}{4}\max \bigl\{ \bigl\vert g'(a) \bigr\vert , \bigl\vert g'(b) \bigr\vert \bigr\} . $$

### Theorem 3

*Let* $g:[a, b]\rightarrow \mathbb{R}$ *be a differentiable function on* $(a, b)$. *If*, *in addition*, *the absolute value function* $\vert g' \vert ^{ \frac{p}{p-1}}$ *is quasiconvex on* $[a, b]$ *with* $p>1$, *then we have the following succeeding inequality*:

3 $$ \biggl\vert \frac{g(a)+g(b)}{2}-\frac{1}{b-a} \int_{a}^{b}g(t)\,dt \biggr\vert \leq \frac{b-a}{2(p+1)^{\frac{1}{p}}} \bigl[ \max \bigl\{ \bigl\vert g'(a) \bigr\vert ^{ \frac{p}{p-1}}, \bigl\vert g'(b) \bigr\vert ^{\frac{p}{p-1}} \bigr\} \bigr] ^{\frac{p-1}{p}}. $$

Subsequently, Alomari et al. [2] obtained the following generalization of Theorem 2.

### Theorem 4

*Let* $g:I\rightarrow \mathbb{R}$ *be a differentiable function on* $I^{\circ }$ *with* $a, b\in I^{\circ }$ *and* $a< b$. *If*, *in addition*, *the absolute value function* $\vert g' \vert ^{q}$ *is quasiconvex on* $[a, b]$, $q\geq 1$, *then we have the following succeeding inequality*:

4 $$ \biggl\vert \frac{g(a)+g(b)}{2}-\frac{1}{b-a} \int_{a}^{b}g(t)\,dt \biggr\vert \leq \frac{b-a}{4} \bigl[\max \bigl\{ \bigl\vert g'(a) \bigr\vert ^{q}, \bigl\vert g'(b) \bigr\vert ^{q}\bigr\} \bigr]^{ \frac{1}{q}}. $$

Recently, Gordji et al. [6] introduced a new class of functions, called the *η*-quasiconvex functions. We present the definition for completeness.

### Definition 5

A function $g:I\subset \mathbb{R}\rightarrow \mathbb{R}$ is said to be an *η*-quasiconvex function with respect to $\eta :\mathbb{R} \times \mathbb{R}\rightarrow \mathbb{R}$ if

$$ g\bigl(tx+(1-t)y\bigr)\leq \max \bigl\{ g(y), g(y)+\eta \bigl(g(x), g(y)\bigr) \bigr\} $$

for all $x, y\in I$ and $t\in [0, 1]$.

For some results concerning the *η*-convex functions and related results, we refer the interested reader to the papers [4, 7, 9, 10, 12, 13, 15–17] and the references therein. Recently, Awan et al. [3] proposed the following definition, which gives a further generalization of Definition 5.

### Definition 6

A function $g:I\subset \mathbb{R}\rightarrow \mathbb{R}$ is said to be a strongly *η*-quasiconvex function with respect to $\eta : \mathbb{R}\times \mathbb{R}\rightarrow \mathbb{R}$ and modulus $\mu \geq 0$ if

$$ g\bigl(tx+(1-t)y\bigr)\leq \max \bigl\{ g(y), g(y)+\eta \bigl(g(x), g(y)\bigr) \bigr\} - \mu t(1-t) (y-x)^{2} $$

for all $x, y\in I$ and $t\in [0, 1]$.

### Example 7

The function $g(x)=x^{2}$ is strongly *η*-quasiconvex with respect to the bifunction $\eta (x, y)=2x+y$ and modulus $\mu =1$. To see this, let $t\in [0, 1]$. Then

$$\begin{aligned} &\max \bigl\{ g(y), g(y)+\eta \bigl(g(x), g(y)\bigr) \bigr\} -\mu t(1-t) (y-x)^{2} \\ &\quad \geq g(y)+\eta \bigl(g(x), g(y)\bigr)- t(1-t) (y-x)^{2} \\ &\quad \geq y^{2}+t\bigl(2x^{2}+y^{2}\bigr)- t(1-t) (y-x)^{2} \\ &\quad =t^{2}x^{2}+2xyt(1-t)+(1-t)^{2}y^{2}+ t\bigl(x^{2}+2y^{2}\bigr) \\ &\quad \geq t^{2}x^{2}+2xyt(1-t)+(1-t)^{2}y^{2} \\ &\quad =g\bigl(tx+(1-t)y\bigr). \end{aligned}$$

### Remark 8

If *g* is strongly *η*-quasiconvex with respect to $\eta (x, y)=x-y$ and modulus $\mu =0$, then Definition 6 reduces to the classical definition of the quasiconvexity.

Our purpose in this paper is to prove analogues of inequalities (1)–(4) for the strongly *η*-quasiconvex functions via the *k*-Riemann–Liouville fractional integral operators. We recapture these inequalities as particular cases of our results (see Remark 20).

We close this section by presenting the definition of the *k*-Riemann–Liouville fractional integral operators.

### Definition 9

(See [11])

The left- and right-sided *k*-Riemann–Liouville fractional integral operators ${}_{k}{\mathbf{{J}}}_{a^{+}}^{\alpha }$ and ${}_{k}{\mathbf{{J}}}_{b^{-}} ^{\alpha }$ of order $\alpha >0$, for a real-valued continuous function $g(x)$, are defined as

5 $$ _{k}{\mathbf{{J}}}_{a^{+}}^{\alpha }g(x) = \frac{1}{k\Gamma_{k}(\alpha )} \int_{a}^{x}(x-t)^{\frac{\alpha }{k}-1}g(t)\,dt, \quad x>a, $$

and

6 $$ _{k}{\mathbf{{J}}}_{b^{-}}^{\alpha }g(x) = \frac{1}{k\Gamma_{k}(\alpha )} \int_{x}^{b} ( t-x ) ^{\frac{\alpha }{k}-1}g(t)\,dt, \quad x< b, $$

where $k>0$, and $\Gamma_{k}$ is the *k*-gamma function given by

$$ \Gamma_{k}(x):= \int_{0}^{\infty }t^{x-1}e^{-\frac{t^{k}}{k}}\,dt, \quad \operatorname{Re}(x)>0, $$

with the properties $\Gamma_{k}(x+k)=x\Gamma_{k}(x)$ and $\Gamma_{k}(k)=1$.

This paper is made up of two sections. In Sect. 2, our main results are framed and justified. Some new inequalities are also obtained as corollaries of the main results.

## Main results

In what follows, we will use the following notation (where convenient): for $g:[a, b]\rightarrow \mathbb{R}$ and $\eta :\mathbb{R}\times \mathbb{R}\rightarrow \mathbb{R}$, we define

$$ \mathcal{M} ( g;\eta ) :=\max \bigl\{ g(b), g(b)+\eta \bigl(g(a), g(b)\bigr) \bigr\} $$

and

$$ \mathcal{N} ( g;\eta ) :=\max \bigl\{ g(a), g(a)+\eta \bigl(g(b), g(a)\bigr) \bigr\} . $$

We now state and prove our first result of this paper.

### Theorem 10

*Let* $\alpha , k>0$, *and let* $g:[a, b]\rightarrow \mathbb{R}$ *be a positive strongly* *η*-*quasiconvex function with modulus* $\mu \geq 0$. *If* $g\in L_{1}([a, b])$, *then we have the following inequality*:

$$\begin{aligned} \frac{\Gamma_{k}(\alpha +k)}{2(b-a)^{\frac{\alpha }{k}}} \bigl[ _{k} {\mathbf{J}}_{a^{+}}^{\alpha }g(b)+ _{k}{\mathbf{J}}_{b^{-}}^{\alpha }g(a) \bigr] \leq \frac{\mathcal{M} ( g;\eta ) +\mathcal{N} ( g;\eta ) }{2}- \alpha \mu (b-a)^{2} \biggl(\frac{1}{\alpha +k}- \frac{1}{\alpha +2k} \biggr). \end{aligned}$$

### Proof

The function *g* is strongly *η*-quasiconvex on $[a, b]$ with $\mu \geq 0$. This implies that

7 $$ \begin{aligned}[b] g\bigl(ta+(1-t)b\bigr) &\leq \max \bigl\{ g(b), g(b)+\eta \bigl(g(a), g(b)\bigr)\bigr\} -\mu t(1-t) (b-a)^{2} \\ &=\mathcal{M} ( g;\eta ) -\mu t(1-t) (b-a)^{2} \end{aligned} $$

and

8 $$ \begin{aligned}[b] g\bigl((1-t)a+tb\bigr) &\leq \max \bigl\{ g(a), g(a)+\eta \bigl(g(b), g(a)\bigr)\bigr\} -\mu t(1-t) (b-a)^{2} \\ &=\mathcal{N} ( g;\eta ) -\mu t(1-t) (b-a)^{2} \end{aligned} $$

for all $t\in [0, 1]$.

By adding (7) and (8) we obtain

9 $$\begin{aligned} &g\bigl(ta+(1-t)b\bigr) +g\bigl((1-t)a+tb\bigr) \\ &\quad \leq \mathcal{M} ( g;\eta ) +\mathcal{N} ( g;\eta ) -2 \mu t(1-t) (b-a)^{2}. \end{aligned}$$

Now, multiplying both sides of (9) by $t^{\frac{\alpha }{k}-1}$ and thereafter integrating the outcome with respect to *t* over the interval $[0, 1]$ give

10 $$\begin{aligned} & \int_{0}^{1}t^{\frac{\alpha }{k}-1}g\bigl(ta+(1-t)b\bigr) \,dt+ \int_{0}^{1}t ^{\frac{\alpha }{k}-1}g\bigl((1-t)a+tb\bigr) \,dt \\ & \quad \leq \mathcal{M} ( g;\eta ) \int_{0}^{1}t^{\frac{\alpha }{k}-1} \,dt + \mathcal{N} ( g;\eta ) \int_{0}^{1}t^{ \frac{\alpha }{k}-1}\,dt-2\mu (b-a)^{2} \int_{0}^{1}t^{ \frac{\alpha }{k}-1}t(1-t)\,dt \\ & \quad =\frac{2k}{\alpha } \biggl[\frac{\mathcal{M} ( g;\eta ) + \mathcal{N} ( g;\eta ) }{2}-\alpha \mu (b-a)^{2} \biggl(\frac{1}{ \alpha +k}-\frac{1}{\alpha +2k} \biggr) \biggr]. \end{aligned}$$

Using the substitutions $x=ta+(1-t)b$ and $y=(1-t)a+tb$ in the definition of the *k*-Riemann–Liouville fractional integrals, we obtain

11 $$ \begin{aligned}[b] \int_{0}^{1}t^{\frac{\alpha }{k}-1}g\bigl(ta+(1-t)b\bigr) \,dt&=\frac{1}{(b-a)^{\frac{ \alpha }{k}}} \int_{a}^{b}(b-x)^{\frac{\alpha }{k}-1}g(x)\,dx\\& = \frac{k \Gamma_{k}(\alpha )}{(b-a)^{\frac{\alpha }{k}}}\times_{k}{\mathbf{J}}_{a ^{+}}^{\alpha }g(b)\end{aligned} $$

and

12 $$ \begin{aligned}[b] \int_{0}^{1}t^{\frac{\alpha }{k}-1}g\bigl((1-t)a+tb\bigr) \,dt&=\frac{1}{(b-a)^{\frac{ \alpha }{k}}} \int_{a}^{b}(y-a)^{\frac{\alpha }{k}-1}g(y)\,dy\\& = \frac{k \Gamma_{k}(\alpha )}{(b-a)^{\frac{\alpha }{k}}}\times_{k}{\mathbf{J}}_{b ^{-}}^{\alpha }g(a).\end{aligned} $$

Employing (11) and (12) in (10), we get

$$\begin{aligned} &\frac{k\Gamma_{k}(\alpha )}{(b-a)^{\frac{\alpha }{k}}} \bigl[ _{k} {\mathbf{J}}_{a^{+}}^{\alpha }g(b)+_{k}{ \mathbf{J}}_{b^{-}}^{\alpha }g(a) \bigr] \\ &\quad \leq \frac{2k}{\alpha } \biggl[\frac{\mathcal{M} ( g;\eta ) + \mathcal{N} ( g;\eta ) }{2}-\alpha \mu (b-a)^{2} \biggl(\frac{1}{ \alpha +k}-\frac{1}{\alpha +2k} \biggr) \biggr]. \end{aligned}$$

Hence the intended inequality is reached. □

Setting $\mu =0$ in Theorem 10, we get the following corollary.

### Corollary 11

*Let* $\alpha , k>0$, *and let* $g:[a, b]\rightarrow \mathbb{R}$ *be a positive strongly* *η*-*quasiconvex function with modulus *0. *If* $g\in L_{1}([a, b])$, *then we have the following inequality*:

13 $$\begin{aligned} \frac{\Gamma_{k}(\alpha +k)}{2(b-a)^{\frac{\alpha }{k}}} \bigl[ _{k} {\mathbf{J}}_{a^{+}}^{\alpha }g(b)+ _{k}{\mathbf{J}}_{b^{-}}^{\alpha }g(a) \bigr] \leq \frac{\mathcal{M} ( g;\eta ) +\mathcal{N} ( g;\eta ) }{2}. \end{aligned}$$

The following lemmas will be useful in the proof of the remaining results of this paper.

### Lemma 12

*Let* $\alpha , k>0$, *and let* $g:[a, b]\rightarrow \mathbb{R}$ *be a differentiable function on the interval* $(a, b)$. *If* $g'\in L_{1}([a, b])$, *then we have the following equality for the* *k*-*fractional integral*:

$$ \begin{gathered} \frac{g(a)+g(b)}{2}-\frac{\Gamma_{k}(\alpha +k)}{2(b-a)^{\frac{\alpha }{k}}} \bigl[ _{k}{ \mathbf{J}}_{a^{+}}^{\alpha }g(b)+ _{k}{ \mathbf{J}}_{b^{-}} ^{\alpha }g(a) \bigr] \\\quad =\frac{b-a}{2} \int_{0}^{1} \bigl[(1-t)^{\frac{ \alpha }{k}}-t^{\frac{\alpha }{k}} \bigr]g' \bigl(ta+(1-t)b \bigr)\,dt.\end{gathered} $$

### Proof

The identity is achieved by setting $s=0$ in [1, Lemma 2.1]. □

### Lemma 13

(See [14, 18])

*If* $\sigma \in (0, 1]$ *and* $0\leq x< y$, *then*

$$ \bigl\vert x^{\sigma }-y^{\sigma } \bigr\vert \leq (y-x)^{\sigma }. $$

### Theorem 14

*Let* $\alpha , k>0$, *and let* $g:[a, b]\rightarrow \mathbb{R}$ *be a differentiable function on* $(a,b)$. *If* $\vert g' \vert $ *is strongly* *η*-*quasiconvex on* $[a, b]$ *with modulus* $\mu \geq 0$ *and* $g'\in L_{1}([a, b])$, *then we have the following inequality*:

$$\begin{aligned} & \biggl\vert \frac{g(a)+g(b)}{2}-\frac{\Gamma_{k}(\alpha +k)}{2(b-a)^{\frac{ \alpha }{k}}} \bigl[ _{k}{ \mathbf{J}}_{a^{+}}^{\alpha }g(b)+ _{k}{ \mathbf{J}}_{b ^{-}}^{\alpha }g(a) \bigr] \biggr\vert \\ &\quad \leq \frac{b-a}{\frac{\alpha }{k}+1} \biggl( 1-\frac{1}{2^{\frac{ \alpha }{k}}} \biggr) \mathcal{M} \bigl( \bigl\vert g' \bigr\vert ;\eta \bigr) \\&\quad\quad{} -\mu (b-a)^{3} \biggl[ \frac{1}{ ( \frac{\alpha }{k}+2 ) } \biggl( 1-\frac{1}{2^{\frac{ \alpha }{k}+1}} \biggr) - \frac{1}{ ( \frac{\alpha }{k}+3 ) } \biggl( 1-\frac{1}{2^{\frac{\alpha }{k}+2}} \biggr) \biggr] . \end{aligned}$$

### Proof

We start by making the following observations: for $t\in [0, 1]$, we obtain

14 $$ (1-t)^{\frac{\alpha }{k}}-t^{\frac{\alpha }{k}} \textstyle\begin{cases} \geq 0,& 0\leq t\leq \frac{1}{2}, \\ < 0,& \frac{1}{2}< t\leq 1, \end{cases} $$

and

15 $$\begin{aligned} \int_{0}^{1} \bigl\vert (1-t)^{\frac{\alpha }{k}}-t^{\frac{\alpha }{k}} \bigr\vert \,dt &= \int_{0}^{\frac{1}{2}} \bigl[ (1-t)^{\frac{\alpha }{k}}-t^{\frac{ \alpha }{k}} \bigr] \,dt + \int_{\frac{1}{2}}^{1} \bigl[ t^{\frac{ \alpha }{k}}-(1-t)^{\frac{\alpha }{k}} \bigr] \,dt \\ &=\frac{2}{ ( \frac{\alpha }{k}+1 ) } \biggl( 1-\frac{1}{2^{\frac{ \alpha }{k}}} \biggr) . \end{aligned}$$

Using a similar line of arguments (as previously), we obtain

16 $$ \int_{0}^{1} t(1-t) \bigl\vert (1-t)^{\frac{\alpha }{k}}-t^{ \frac{\alpha }{k}} \bigr\vert \,dt= \frac{2}{ ( \frac{\alpha }{k}+2 ) } \biggl( 1-\frac{1}{2^{\frac{ \alpha }{k}+1}} \biggr) -\frac{2}{ ( \frac{\alpha }{k}+3 ) } \biggl( 1- \frac{1}{2^{\frac{\alpha }{k}+2}} \biggr) . $$

Now, using the fact that $\vert g' \vert $ is strongly *η*-quasiconvex with $\mu \geq 0$ and then applying Lemma 12, the properties of the modulus, and identities (15) and (16), we obtain:

$$\begin{aligned} & \biggl\vert \frac{g(a)+g(b)}{2}-\frac{\Gamma_{k}(\alpha +k)}{2(b-a)^{\frac{ \alpha }{k}}} \bigl[ _{k}{ \mathbf{J}}_{a^{+}}^{\alpha }g(b)+ _{k}{ \mathbf{J}}_{b ^{-}}^{\alpha }g(a) \bigr] \biggr\vert \\ &\quad \leq \frac{b-a}{2} \int_{0}^{1} \bigl\vert (1-t)^{\frac{\alpha }{k}}-t^{\frac{ \alpha }{k}} \bigr\vert \bigl\vert g' \bigl(ta+(1-t)b \bigr) \bigr\vert \,dt \\ &\quad \leq \frac{b-a}{2} \int_{0}^{1} \bigl\vert (1-t)^{\frac{\alpha }{k}}-t^{\frac{ \alpha }{k}} \bigr\vert \bigl[ \max \bigl\{ \bigl\vert g'(b) \bigr\vert , \bigl\vert g'(b) \bigr\vert +\eta \bigl( \bigl\vert g'(a) \bigr\vert , \bigl\vert g'(b) \bigr\vert \bigr) \bigr\} \\ &\quad\quad{} - \mu t(1-t) (b-a)^{2} \bigr] \,dt \\ &\quad =\frac{b-a}{2}\max \bigl\{ \bigl\vert g'(b) \bigr\vert , \bigl\vert g'(b) \bigr\vert +\eta \bigl( \bigl\vert g'(a) \bigr\vert , \bigl\vert g'(b) \bigr\vert \bigr) \bigr\} \int_{0}^{1} \bigl\vert (1-t)^{\frac{\alpha }{k}}-t^{\frac{\alpha }{k}} \bigr\vert \,dt \\ &\quad\quad {} -\mu \frac{(b-a)^{3}}{2} \int_{0}^{1}t(1-t) \bigl\vert (1-t)^{ \frac{\alpha }{k}}-t^{\frac{\alpha }{k}} \bigr\vert \,dt \\ &\quad =\frac{b-a}{2}\max \bigl\{ \bigl\vert g'(b) \bigr\vert , \bigl\vert g'(b) \bigr\vert +\eta \bigl( \bigl\vert g'(a) \bigr\vert , \bigl\vert g'(b) \bigr\vert \bigr) \bigr\} \frac{2}{ ( \frac{\alpha }{k}+1 ) } \biggl( 1-\frac{1}{2^{ \frac{\alpha }{k}}} \biggr) \\ &\quad\quad {} -\mu \frac{(b-a)^{3}}{2} \biggl[ \frac{2}{ ( \frac{\alpha }{k}+2 ) } \biggl( 1- \frac{1}{2^{\frac{\alpha }{k}+1}} \biggr) -\frac{2}{ ( \frac{ \alpha }{k}+3 ) } \biggl( 1-\frac{1}{2^{\frac{\alpha }{k}+2}} \biggr) \biggr] . \end{aligned}$$

Hence the result follows. □

Putting $\mu =0$ in Theorem 14, we obtain the following result.

### Corollary 15

*Let* $\alpha , k>0$, *and let* $g:[a, b]\rightarrow \mathbb{R}$ *be a differentiable function on* $(a,b)$. *If* $\vert g' \vert $ *is strongly* *η*-*quasiconvex on* $[a, b]$ *with modulus* 0 *and* $g'\in L_{1}([a, b])$, *then we have the following inequality*:

17 $$\begin{aligned} & \biggl\vert \frac{g(a)+g(b)}{2}-\frac{\Gamma_{k}(\alpha +k)}{2(b-a)^{\frac{ \alpha }{k}}} \bigl[ _{k}{\mathbf{J}}_{a^{+}}^{\alpha }g(b)+ _{k}{\mathbf{J}}_{b ^{-}}^{\alpha }g(a) \bigr] \biggr\vert \\ &\quad \leq \frac{b-a}{\frac{\alpha }{k}+1} \biggl( 1-\frac{1}{2^{\frac{ \alpha }{k}}} \biggr) \max \bigl\{ \bigl\vert g'(b) \bigr\vert , \bigl\vert g'(b) \bigr\vert +\eta \bigl( \bigl\vert g'(a) \bigr\vert , \bigl\vert g'(b) \bigr\vert \bigr) \bigr\} . \end{aligned}$$

### Theorem 16

*Let* $\alpha , k>0$, $q>1$, *and let* $g:[a, b]\rightarrow \mathbb{R}$ *be a differentiable function on* $(a,b)$. *If* $\vert g' \vert ^{q}$ *is strongly* *η*-*quasiconvex on* $[a, b]$ *with modulus* $\mu \geq 0$ *and* $g'\in L_{1}([a, b])$, *then we have the following inequality*:

$$\begin{aligned} & \biggl\vert \frac{g(a)+g(b)}{2}-\frac{\Gamma_{k}(\alpha +k)}{2(b-a)^{\frac{ \alpha }{k}}} \bigl[ _{k}{ \mathbf{J}}_{a^{+}}^{\alpha }g(b)+ _{k}{ \mathbf{J}}_{b ^{-}}^{\alpha }g(a) \bigr] \biggr\vert \\ &\quad \leq \frac{b-a}{2} \biggl( \frac{1}{{\frac{\alpha }{k}p}+1} \biggr) ^{ \frac{1}{p}} \biggl( \mathcal{M} \bigl( \bigl\vert g' \bigr\vert ^{q};\eta \bigr) -\mu \frac{(b-a)^{2}}{6} \biggr) ^{\frac{1}{q}}, \end{aligned}$$

*where* $\frac{1}{p}+\frac{1}{q}=1$ *and* $\frac{\alpha }{k}\in (0, 1]$.

### Proof

As a consequence of Lemma 13, we have that

$$ \bigl\vert x^{\frac{\alpha }{k}}-y^{\frac{\alpha }{k}} \bigr\vert \leq \vert x-y \vert ^{\frac{ \alpha }{k}} $$

for all $x,y\in [0, 1]$ with $\frac{\alpha }{k}\in (0, 1]$. Using the above information, we make the following computations:

18 $$\begin{aligned} \int_{0}^{1} \bigl\vert (1-t)^{\frac{\alpha }{k}}-t^{\frac{\alpha }{k}} \bigr\vert ^{p}\,dt &\leq \int_{0}^{1} \vert 1-2t \vert ^{\frac{\alpha }{k}p} \,dt \\ &= \int_{0}^{\frac{1}{2}} \vert 1-2t \vert ^{\frac{\alpha }{k}p} \,dt + \int_{\frac{1}{2}}^{1} \vert 1-2t \vert ^{\frac{\alpha }{k}p} \,dt \\ &= \int_{0}^{\frac{1}{2}}(1-2t)^{\frac{\alpha }{k}p}\,dt + \int_{\frac{1}{2}}^{1}(2t-1)^{\frac{\alpha }{k}p}\,dt \\ &=\frac{1}{{\frac{\alpha }{k}p}+1}. \end{aligned}$$

Since the function $\vert g' \vert ^{q}$ is strongly *η*-quasiconvex on $[a, b]$ with modulus $\mu \geq 0$, we have

19 $$ \begin{aligned}[b] \bigl\vert g'\bigl(ta+(1-t)b\bigr) \bigr\vert ^{q}&\leq \max \bigl\{ \bigl\vert g'(b) \bigr\vert ^{q}, \bigl\vert g'(b) \bigr\vert ^{q}+\eta \bigl( \bigl\vert g'(a) \bigr\vert ^{q}, \bigl\vert g'(b) \bigr\vert ^{q}\bigr) \bigr\} \\ &\quad{} -\mu t(1-t) (b-a)^{2}.\end{aligned} $$

Now, applying Lemma 12, the Hölder inequality, the properties of absolute values, and inequalities (18) and (19), we obtain

$$\begin{aligned} & \biggl\vert \frac{g(a)+g(b)}{2}-\frac{\Gamma_{k}(\alpha +k)}{2(b-a)^{\frac{ \alpha }{k}}} \bigl[ _{k}{ \mathbf{J}}_{a^{+}}^{\alpha }g(b)+ _{k}{ \mathbf{J}}_{b ^{-}}^{\alpha }g(a) \bigr] \biggr\vert \\ &\quad \leq \frac{b-a}{2} \int_{0}^{1} \bigl\vert (1-t)^{\frac{\alpha }{k}}-t^{\frac{ \alpha }{k}} \bigr\vert \bigl\vert g' \bigl(ta+(1-t)b \bigr) \bigr\vert \,dt \\ &\quad \leq \frac{b-a}{2} \biggl( \int_{0}^{1} \bigl\vert (1-t)^{\frac{\alpha }{k}}-t ^{\frac{\alpha }{k}} \bigr\vert ^{p}\,dt \biggr) ^{\frac{1}{p}} \biggl( \int_{0} ^{1} \bigl\vert g' \bigl(ta+(1-t)b \bigr) \bigr\vert ^{q}\,dt \biggr) ^{\frac{1}{q}} \\ &\quad \leq \frac{b-a}{2} \biggl( \frac{1}{{\frac{\alpha }{k}p}+1} \biggr) ^{ \frac{1}{p}} \biggl( \int_{0}^{1} \bigl[ \max \bigl\{ \bigl\vert g'(b) \bigr\vert ^{q}, \bigl\vert g'(b) \bigr\vert ^{q}+ \eta \bigl( \bigl\vert g'(a) \bigr\vert ^{q}, \bigl\vert g'(b) \bigr\vert ^{q}\bigr) \bigr\} \\ &\quad\quad{} -\mu t(1-t) (b-a)^{2} \bigr] \,dt \biggr) ^{\frac{1}{q}} \\ &\quad =\frac{b-a}{2} \biggl( \frac{1}{{\frac{\alpha }{k}p}+1} \biggr) ^{ \frac{1}{p}} \biggl( \max \bigl\{ \bigl\vert g'(b) \bigr\vert ^{q}, \bigl\vert g'(b) \bigr\vert ^{q}+\eta \bigl( \bigl\vert g'(a) \bigr\vert ^{q}, \bigl\vert g'(b) \bigr\vert ^{q}\bigr) \bigr\} -\mu \frac{(b-a)^{2}}{6} \biggr) ^{\frac{1}{q}}. \end{aligned}$$

This completes the proof. □

Taking $\mu =0$ in Theorem 16, we get the following:

### Corollary 17

*Let* $\alpha , k>0$, $q>1$, *and let* $g:[a, b]\rightarrow \mathbb{R}$ *be a differentiable function on* $(a,b)$. *If* $\vert g' \vert ^{q}$ *is strongly* *η*-*quasiconvex on* $[a, b]$ *with modulus* 0 *and* $g'\in L_{1}([a, b])$, *then we have the following inequality*:

20 $$\begin{aligned} & \biggl\vert \frac{g(a)+g(b)}{2}-\frac{\Gamma_{k}(\alpha +k)}{2(b-a)^{\frac{ \alpha }{k}}} \bigl[ _{k}{\mathbf{J}}_{a^{+}}^{\alpha }g(b)+ _{k}{\mathbf{J}}_{b ^{-}}^{\alpha }g(a) \bigr] \biggr\vert \\ &\quad \leq \frac{b-a}{2} \biggl( \frac{1}{{\frac{\alpha }{k}p}+1} \biggr) ^{ \frac{1}{p}} \bigl(\max \bigl\{ \bigl\vert g'(b) \bigr\vert ^{q}, \bigl\vert g'(b) \bigr\vert ^{q}+\eta \bigl( \bigl\vert g'(a) \bigr\vert ^{q}, \bigl\vert g'(b) \bigr\vert ^{q}\bigr) \bigr\} \bigr)^{\frac{1}{q}}, \end{aligned}$$

*where* $\frac{1}{p}+\frac{1}{q}=1$ *and* $\frac{\alpha }{k}\in (0, 1]$.

Finally, we present the following result.

### Theorem 18

*Let* $\alpha , k>0$, $q\geq 1$, *and let* $g:[a, b]\rightarrow \mathbb{R}$ *be a differentiable function on* $(a,b)$. *If* $\vert g' \vert ^{q}$ *is strongly* *η*-*quasiconvex on* $[a, b]$ *with modulus* $\mu \geq 0$ *and* $g'\in L_{1}([a, b])$, *then we have the following inequality*:

$$\begin{aligned} & \biggl\vert \frac{g(a)+g(b)}{2}-\frac{\Gamma_{k}(\alpha +k)}{2(b-a)^{\frac{ \alpha }{k}}} \bigl[ _{k}{ \mathbf{J}}_{a^{+}}^{\alpha }g(b)+ _{k}{ \mathbf{J}}_{b ^{-}}^{\alpha }g(a) \bigr] \biggr\vert \\ &\quad \leq \frac{b-a}{2} \bigl(\mathcal{P}(\alpha ;k) \bigr)^{1-\frac{1}{q}} \bigl(\mathcal{M} \bigl( \bigl\vert g' \bigr\vert ^{q}; \eta \bigr) \mathcal{P}(\alpha ;k)- \mu (b-a)^{2}\mathcal{Q}(\alpha ;k) \bigr)^{\frac{1}{q}}, \end{aligned}$$

*where*

$$ \mathcal{P}(\alpha ;k)=\frac{2}{ ( \frac{\alpha }{k}+1 ) } \biggl( 1-\frac{1}{2^{\frac{\alpha }{k}}} \biggr) $$

*and*

$$ \mathcal{Q}(\alpha ;k)=\frac{2}{ ( \frac{\alpha }{k}+2 ) } \biggl( 1-\frac{1}{2^{\frac{\alpha }{k}+1}} \biggr) - \frac{2}{ ( \frac{ \alpha }{k}+3 ) } \biggl( 1-\frac{1}{2^{\frac{\alpha }{k}+2}} \biggr) . $$

### Proof

We follow similar arguments as in the proof of the previous theorem. For this, we use again Lemma 12, the Hölder inequality, and the properties of the absolute values to obtain

$$\begin{aligned} & \biggl\vert \frac{g(a)+g(b)}{2}-\frac{\Gamma_{k}(\alpha +k)}{2(b-a)^{\frac{ \alpha }{k}}} \bigl[ _{k}{ \mathbf{J}}_{a^{+}}^{\alpha }g(b)+ _{k}{ \mathbf{J}}_{b ^{-}}^{\alpha }g(a) \bigr] \biggr\vert \\ &\quad \leq \frac{b-a}{2} \int_{0}^{1} \bigl\vert (1-t)^{\frac{\alpha }{k}}-t^{\frac{ \alpha }{k}} \bigr\vert \bigl\vert g' \bigl(ta+(1-t)b \bigr) \bigr\vert \,dt \\ &\quad \leq \frac{b-a}{2} \biggl( \int_{0}^{1} \bigl\vert (1-t)^{\frac{\alpha }{k}}-t ^{\frac{\alpha }{k}} \bigr\vert \,dt \biggr) ^{1-\frac{1}{q}} \biggl( \int_{0} ^{1} \bigl\vert (1-t)^{\frac{\alpha }{k}}-t^{\frac{\alpha }{k}} \bigr\vert \bigl\vert g' \bigl(ta+(1-t)b \bigr) \bigr\vert ^{q}\,dt \biggr) ^{\frac{1}{q}} \\ &\quad \leq \frac{b-a}{2} \biggl( \int_{0}^{1} \bigl\vert (1-t)^{\frac{\alpha }{k}}-t ^{\frac{\alpha }{k}} \bigr\vert \,dt \biggr) ^{1-\frac{1}{q}} \\ &\quad \quad {} \times \biggl( \int_{0}^{1} \bigl\vert (1-t)^{\frac{\alpha }{k}}-t^{\frac{ \alpha }{k}} \bigr\vert \bigl[ \max \bigl\{ \bigl\vert g'(b) \bigr\vert ^{q}, \bigl\vert g'(b) \bigr\vert ^{q}+\eta \bigl( \bigl\vert g'(a) \bigr\vert ^{q}, \bigl\vert g'(b) \bigr\vert ^{q}\bigr) \bigr\} \\ &\quad\quad{} -\mu t(1-t) (b-a)^{2} \bigr] \,dt \biggr) ^{\frac{1}{q}}. \end{aligned}$$

The desired inequality follows by appealing to identities (15) and (16). □

Taking $\mu =0$ in Theorem 18, we get the succeeding corollary.

### Corollary 19

*Let* $\alpha , k>0$, $q\geq 1$, *and let* $g:[a, b]\rightarrow \mathbb{R}$ *be a differentiable function on* $(a,b)$. *If* $\vert g' \vert ^{q}$ *is strongly* *η*-*quasiconvex on* $[a, b]$ *with modulus* 0 *and* $g'\in L_{1}([a, b])$, *then we have the following inequality*:

21 $$\begin{aligned} & \biggl\vert \frac{g(a)+g(b)}{2}-\frac{\Gamma_{k}(\alpha +k)}{2(b-a)^{\frac{ \alpha }{k}}} \bigl[ _{k}{\mathbf{J}}_{a^{+}}^{\alpha }g(b)+ _{k}{\mathbf{J}}_{b ^{-}}^{\alpha }g(a) \bigr] \biggr\vert \\ &\quad \leq \frac{b-a}{2}\mathcal{P}(\alpha ;k) \bigl(\max \bigl\{ \bigl\vert g'(b) \bigr\vert ^{q}, \bigl\vert g'(b) \bigr\vert ^{q}+\eta \bigl( \bigl\vert g'(a) \bigr\vert ^{q}, \bigl\vert g'(b) \bigr\vert ^{q}\bigr) \bigr\} \bigr)^{\frac{1}{q}}, \end{aligned}$$

*where*

$$ \mathcal{P}(\alpha ;k)=\frac{2}{ ( \frac{\alpha }{k}+1 ) } \biggl( 1-\frac{1}{2^{\frac{\alpha }{k}}} \biggr) . $$

### Remark 20

Substituting $\eta (x,y)=x-y$ and $\alpha =k=1$ into (13), (17), (20), and (21), we recover (1), (2), (3), and (4), respectively.

## Conclusion

Four main results of the Hermite–Hadamard kind for functions that are strongly *η*-quasiconvex with modulus $\mu \geq 0$ are hereby established. We recover known results in the literature by setting $\eta (x,y)=x-y$, $\alpha =k=1$, and $\mu =0$ in Theorems 10, 14, 16, and 18. More results can be obtained by choosing different bifunction *η* and then *μ*.
